# COVID-19-related dysfunctional anxiety and associated factors among adolescents in Southwest Ethiopia: a cross-sectional study

**DOI:** 10.1186/s12888-024-05587-5

**Published:** 2024-02-27

**Authors:** Robera Demissie Berhanu, Jira Wakoya Feyisa, Jibril Dori Boru, Desalegn Emana Jabana, Birbirsa Sefera Senbeta, Million Girma Tekle, Yadeta Alemayehu, Hunde Tarafa Aga

**Affiliations:** 1 School of Nursing and Midwifery, Institute of Health Sciences, Wallaga University, Nekemte, Ethiopia; 2 Department of Public Health, Institute of Health Sciences, Wallaga University, Nekemte, Ethiopia; 3 Department of Pharmacy, College of Health Sciences, Mattu University, Mettu, Ethiopia; 4 Department of Psychiatry, College of Health Sciences, Mattu University, Mettu, Ethiopia

**Keywords:** COVID-19, Dysfunctional anxiety, Adolescents, Mental health

## Abstract

**Background:**

COVID-19 pandemic causes serious threats to people’s mental health, particularly it has huge negative mental health outcomes for adolescents. However, there is lack of studies examining COVID-19-related anxiety among adolescents in Ethiopia. Hence, this study was aimed to examine COVID-19-related dysfunctional anxiety and its associated factors among adolescents in Mettu town.

**Methods:**

Community-based cross-sectional study was conducted from September 1 to 30, 2020 among 847 adolescents selected by stratified sampling technique. IBM SPSS Statistics Version 26.0 was used for analysis. Descriptive statistics such as frequency, percentage, mean, and standard deviation were computed. Bivariate and multivariate binary logistic regression analyses were done to identify factors associated with COVID-19-related dysfunctional anxiety. The statistical significance was declared at *p* ≤ 0.05; and the strength of association was described in terms of adjusted odds ratio.

**Results:**

Out of the total sample, 819 adolescents participated in this study. The mean age of the participants was 14.9 (SD = 2.798) years. The magnitude of COVID-19-related dysfunctional anxiety was found to be 20.9% (95% CI (18.1, 23.9)). The finding indicates that sex [(AOR (95% CI)); (0.724 (0.502, 1.043))], having both parents deceased [(AOR (95% CI)); (2.981 (1.138, 7.814))], living alone [(AOR (95% CI)); (2.363 (1.050, 5.321))], having unemployed mothers [(AOR (95% CI)); (1.943 (1.194, 3.163))], absence of close friend [(AOR (95% CI)); (0.377 (0.225, 0.630))], and medical problem [(AOR (95% CI)); (0.408 (0.278, 0.597))] were significantly associated with COVID-19-related anxiety.

**Conclusion:**

The magnitude of COVID-19-related dysfunctional anxiety was found to be high in the study area. The findings have shown that the likelihood of developing COVID-19-related dysfunctional anxiety was linked to several factors. Provision of continued psychological support for adolescents is extremely encouraged.

## Background

The Coronavirus Disease 2019 (COVID-19) is a global pandemic that first appeared in China in late December 2019 and spread throughout the world in early 2020. It is caused by severe acute respiratory syndrome coronavirus 2 (SARS-CoV-2) [1]. In Africa, the first case of COVID-19 was reported in Egypt on 14^th^ February 2020 [2]. The first case was reported in Ethiopia on 13^th^ March 2020 [3]. More than 54 million cases and 1.3 million fatalities have been caused by the virus globally [4]. Because of the sudden nature of the pandemic and the virus’s ability to spread quickly, the pandemic causes serious threats to people’s physical and mental health. Many psychological problems, such as panic disorder, anxiety, depression, and stress, have been triggered by the pandemic [1, 5, 6]. Evidence indicates that COVID-19 patients might encounter a wide range of mental health problems, including anxiety, depression, insomnia, and delirium [6]. The pandemic could induce those mental and behavioural disorders either directly through viral infection of the central nervous system or indirectly via initiating immune response [7]. Different studies demonstrated that the virus is potentially neurotropic and can induce neuronal injuries [8]. In spite of the possible brain infiltration, “cytokines storm” involved in the immune response to SARS-CoV-2 may cause psychiatric symptoms by precipitating inflammatory response within the brain [9, 10]. In addition to these, fear of illness, uncertainty of the future, stigma, traumatic memories of severe illness, and social isolation experienced by adolescents during the COVID-19 are significant psychological stressors [11, 12]. Therefore, examining COVID-19-related dysfunctional anxiety among adolescents in a country like Ethiopia where mental health is given less concern and surrounded by stigmatization is extremely crucial for improving mental health for adolescents.

Based on WHO age classification, adolescents are individuals who are in the age group of 10 to 19 years [13]. These individuals have highly destabilizing and psychological effects because of many reasons despite the fact that psychological and emotional reactions at this stage are less focused. Adolescence is a period of storm and stress [14]. This developmental stage is characterized by increased emotionality in response to actual and/or perceived stressors due to neural mismatch owing to physical and chemical changes in the brain [15], yet the self-regulatory system required to manage these emotions remains largely underdeveloped until early adulthood [16]. Additionally, profoundly increased social sensitivity and the significance of peers characterizes adolescence [17]. During this period, adolescents strive for independence from their parents, start spending much of their time with peers, and choose friends rather than parents as the primary source of interaction [18]. It is shown that creation of positive relationship with peers at this stage helps adolescents as it could provide social and emotional support, and this in turn protects them against the risk of developing anxiety and depression [19]. But such important relationships and sociability might be compromised by sudden and serious danger [20], as it is true with COVID-19 pandemic.

COVID-19-related dysfunctional anxiety is triggered by public health measures taken to stop the spread of the SARS-CoV-2 virus. To mitigate the risks and impacts of the COVID-19 pandemic, the local government has adopted a variety of public health measures, such as social distancing, school suspensions, and the shutdown of non-essential services. Such abrupt daily life transformations could be another reason for profound consequences on mental health, including anxiety [11, 21]. While these lifestyle transformations are challenging for people of all ages, they may be particularly difficult for adolescents who rely heavily on their peer connections for emotional support and social development [22]. These changes may cause depressive and anxious disorders. Without appropriate psychological interventions, depression and anxiety among adolescents often persist into adulthood and elevate the risk factors of age-related disease, such as cardiovascular diseases [23, 24]. As these lifestyle transformations impede most peer interaction, it is important to examine the magnitude of COVID-19-related dysfunctional anxiety and its associated factors among adolescents. However, to date, there is no research that evaluated COVID-19-related dysfunctional anxiety among adolescents in Ethiopia, particularly in the study area even though there are several studies conducted on different groups of population. Therefore, the goal of this study was to assess COVID-19-related dysfunctional anxiety and its associated factors among adolescents in Mettu town. Hence, the findings from this study will shed light on the magnitude of COVID-related dysfunctional anxiety among adolescents and the factors that contribute to it. It will provide an input and contribution to various health and education sectors in designing interventions and strategies to enhance mental health of adolescents as well as in preparation for the future pandemic.

## Materials and methods

### Study design, setting, and period

A community-based cross-sectional study was conducted in Mettu town from September 1 to 30, 2020 among 847 adolescents. Mettu town is the capital town of the Ilu Abba Bor Zone, Oromia regional state. The town is located at 600 km to the southwest of Addis Ababa, the country’s capital. The town has three administrative kebeles (kebeles are the lowest administrative unit in Ethiopia), namely Soor, Abba mole, and Kollo korma kebeles. The town has an estimated total adolescent population of 21,844. The total number of households found in the town is 22,682, with 7541 households in Soor Kebele, 7937 households in Abba mole kebele, and 7204 households in Kollo korma kebele.

### Study population and recruitment criteria

The study population for this study was all adolescents in Mettu town who fulfilled eligibility criteria. All adolescents who were residents of Mettu town for at least six months were included in this study and those who were critically ill during the data collection period and/or who were unable to communicate were excluded.

### Sampling procedure

Stratified sampling technique was used to select participants for the study. Preliminary survey was done to identify and code households containing adolescents. Accordingly, 4191, 4112, and 4250 households were identified and coded from Soor, Abba mole, and Kollo korma kebeles, respectively. Then the overall sample size was proportionally allocated to each kebele based on the size of households containing adolescents in the kebeles. Finally, simple random sampling was used to select households from the identified and coded households containing adolescents. Adolescent within each selected household was interviewed. Kish grid method was used to determine which adolescent to interview where more than one eligible adolescent was encountered in the selected household [25].

### Study instruments and measurement of variables

Interviewer-administered structured questionnaire was used to collect data. The questionnaire had several categories: (a) socio-demographic characteristics, (b) family characteristics, (c) psychosocial and health related factors, (d) Oslo 3-items social support scale, (e) fear of COVID-19 scale, and (f) COVID-19 anxiety scale (CAS). Oslo 3-items social support scale was used to measure social support. The items were added together and it yielded a total score ranging from 3 to 14 that was interpreted as score below the mean value is poor social support, and the score equals to mean score or above is strong social support [26]. In this study, Cronbach’s alpha for Oslo 3-items social support scale is 0.83. Fear of COVID-19 scale was used to measure adolescents’ fear of COVID-19 infection. The scale has 5 items and the participants rated their level of agreement on 5-point Likert scale. Each item was added up to produce the total score ranging from 7 to 35. The values equal to or greater than the mean score were considered as high fear of COVID-19 infection, while the rest were considered low fear [27, 28]. The Cronbach’s alpha for fear of COVID-19 scale is found to be 0.78 in this study. CAS was used to measure COVID-19-related dysfunctional anxiety, and it is a self-report mental health screener of dysfunctional anxiety associated with the coronavirus crisis. CAS has 5 items that are rated on a 5-point scale, from 0 (not at all) to 4 (nearly every day), based on experiences over the past 2 weeks. The total scale was calculated by adding all the items together produceing the score ranging from 5 to 25. Therefore, COVID-19-related dysfunctional anxiety is harmful anxiety related to COVID-19 measured on 5-point Likert scale with the scores ranging from 5 to 25 and the values greater than or equal to 9 indicate presence of COVID-19-related dysfunctional anxiety [29]. The Cronbach’s alpha for CAS was 0.84 in a study conducted in Bangladesh and 0.856 in a study conducted in Ethiopia among health professionals. The Cronbach’s alpha for CAS is 0.89 in the current study.

### Data collection and data quality control

Data were collected by five B.Sc. nurses under the supervision of two B.Sc. psychiatric nurses. The data collectors approached the study participants by strictly following COVID-19 prevention precautions, such as wearing masks and standing 1 m away. Pre-test was conducted on 43 adolescents at Gore town; and then vague and ambiguous questions were revised and modified based on the pre-test findings. Data collectors and supervisors were trained for one day on the procedures of data collection, informed consent or assent, maintaining the privacy, and infection prevention mechanisms related to COVID-19. Eligible participants not found on the day of data collection were revisited three times at different time intervals. Finally, the collected data were checked for completeness.

### Statistical analysis

Data were coded and entered using Epi-data manager version 3.1 and then exported to IBM SPSS Statistics Version 26.0 for analysis. Descriptive statistics such as frequency, percentage, mean, and standard deviation were computed. Bivariate binary logistic regression analysis was done to select candidate variables for the final model. Based on the recommendations of the Hosmer Jr, Lemeshow, & Sturdivant [30], *p* < 0.25 cut off point and theoretical knowledge were used to select candidate variables to be entered into the multivariable logistic regression model. The fitness of the multivariable logistic regression model was checked by Hosmer and Lemeshow’s test, which was found to be insignificant (*p* = 0.115). Finally, the statistical significance was declared at *p* ≤ 0.05; and the strength of association was described in terms of adjusted odds ratio (AOR) with respective 95% confidence interval (CI).

## Results

### Socio-demographic characteristics

Out of the total sample, 819 adolescents participated in the study giving a response rate of 96.7%. Nearly half (51.3%) of participants were female. The mean age of the participants was 14.9 (SD = 2.798) years. Three-quarters of the respondents (75%) were Oromo and more than one-third (37.7%) of them were orthodox in religion. The vast majority (93.8%) of adolescents participated in this study had formal education (Table 1).

**Table 1:** Socio-demographic characteristics of adolescents in Mettu town, Southwest Ethiopia, September 2020 (*n* = 819)

Variables	Categories	Frequency	Percentage (%)
**Sex**	Male	399	48.7
	Female	420	51.3
**Age**	10 – 14	365	44.6
	15 – 19	454	55.4
**Religion**	Protestant	242	29.5
	Orthodox	309	37.7
	Muslim	240	29.3
	Wakefata	28	3.5
**Ethnicity**	Oromo	614	75.0
	Amhara	100	12.2
	Tigre	67	8.2
	Others^a^	38	4.6
**Education**	No formal education	51	6.2
	Has formal education	768	93.8
**Living status**	Lives with parents or other	787	96.1
	Lives alone	32	3.9

^a^Kaffa, Wolaita, Silte and Agnuak

### Family characteristics

For most of the adolescents (87.1%), both fathers and mothers are alive, while 2.7% of them stated that both father and mother were deceased. Most of the participants’ fathers (71.1%) and mothers (76.6%) were unemployed. Most of the participants’ fathers (81.3%) and mothers (77%) had formal education. Most of the adolescents involved (63.1%) in this study were from family who earn < 2143 ETB on average per month (Table 2).

**Table 2:** Family characteristics of adolescents in Mettu town, Southwest Ethiopia, September 2020 (*n* = 819)

Variables	Categories	Frequency	Percentage (%)
**Parents alive?**	Both are alive	713	87.1
	Only mother is alive	54	6.6
	Only father is alive	30	3.7
	Both are deceased	22	2.7
**Father’s occupation**	Employed	237	28.9
	Unemployed	582	71.1
**Mother’s occupation**	Employed	192	23.4
	Unemployed	627	76.6
**Father’s educational status**	No formal education	153	18.7
	Has formal education	666	81.3
**Mother’s educational status**	No formal education	188	23
	Has formal education	631	77
**Average family monthly income**	< 2143 ETB	517	63.1
	≥ 2143 ETB	302	36.9

*ETB* Ethiopian birr

### Psychosocial and health related factors

Many of the participants (88.8%) reported that they had close friends. Nearly one-third (37.4%) of the participants stated that they had no known medical problem. About one-tenth (9.6%) of the participants stated that they had family history of mental illness. About one-fourth (24.5%) of the participants reported that they used substance in the last 3 months. Khat was the most commonly used substance (21.4%) according to the participants’ reports (Table 3).

**Table 3:** Psychosocial and health related factors of adolescents in Mettu town, Southwest Ethiopia, September 2020 (*n* = 819)

Variables	Categories	Frequency	Percentage (%)
**Close friend**	Has no close friend	92	11.2
	Has close friend	727	88.8
**Have known medical problem**	Yes	513	62.6
	No	306	37.4
**Family history of mental illness**	Yes	81	9.6
	No	738	90.4
**Substance use in the last 3 months**	Yes	201	24.5
	No	618	75.5
**Type of substance used**	Khat	175	21.4
	Cigarette	65	7.9
	Alcohol	82	10
	Other	16	4.7
**Parents use of substance in the past 3 months**	Yes	55	6.6
	No	765	93.4

### Psychological distress, fear of COVID-19, and social support

**Fig. 1:**
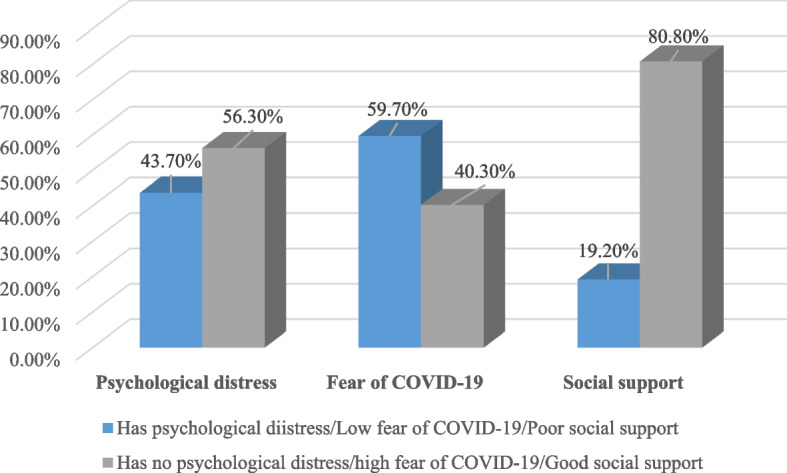
Psychological distress, fear of COVID-19, and social support among adolescents in Mettu town, Southwest Ethiopia, September 2020 (*n* = 819)

Out of the participants involved in this study, 43.7% of them had psychological distress, 59.7% had high fear of COVID-19, and 19.2% had poor social support (Fig. 1).

### COVID-19-related anxiety

**Fig. 2:**
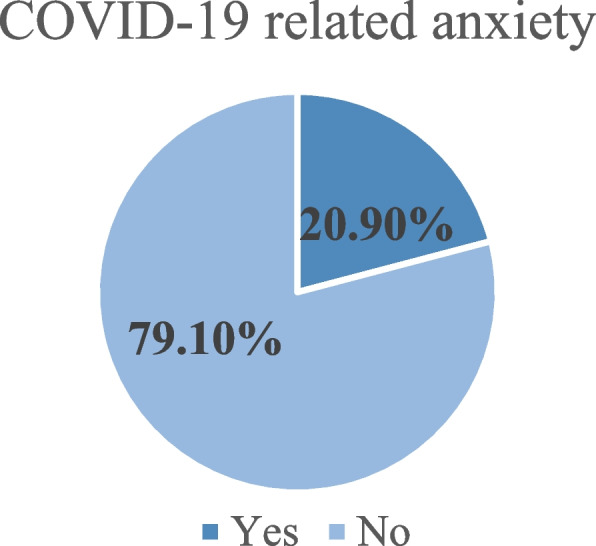
COVID-19-related dysfunctional anxiety among adolescents in Mettu town, Southwest Ethiopia, September 2020 (*n* = 819)

Out of the participants involved in this study, 171 (20.9%) of them had COVID-19-related dysfunctional anxiety (95% CI (18.1, 23.9)) (Fig. 2).

### Factors associated with COVID-19-related anxiety

Bivariate binary logistic regression analyses were used to select candidate variables for multivariable linear regression analysis. After the analyses were carried out, 13 variables were selected and entered into multivariable binary logistic regression model using backward elimination to see their association with COVID-19-related anxiety. From variables entered into the final model, the multivariable binary logistic regression model revealed that sex, parents alive, living status, mother’s occupation, psychological distress, presence of close friend, and substance use were significantly associated with COVID-19-related anxiety.

Based on the findings, the odds of COVID-19-related dysfunctional anxiety was decreased by 31.5% among female adolescents when compared to male adolescents [AOR (95% CI)], [0.724 (0.502, 1.043)]. Similarly, the odds of COVID-19-related dysfunctional anxiety was 2.853 times higher among adolescents who reported that both parents were deceased than those who reported either both or one were/was alive [AOR (95% CI)], [2.981 (1.138, 7.814)]. Furthermore, the odds of COVID-19-related dysfunctional anxiety was 2.311 times higher among adolescents who reported living alone than those who reported living with their parents or with other people alive [AOR (95% CI)], [2.363 (1.050, 5.321)]. The odds of COVID-19-related dysfunctional anxiety was 1.919 times higher among adolescents whose mothers were unemployed compared to those who had employed mothers [AOR (95% CI)], [1.943 (1.194, 3.163)]. The odds of COVID-19-related dysfunctional anxiety was reduced by 60.1% among adolescents who reported to have close friend(s) compared to those who reported not having close friend [AOR (95% CI)], [0.377 (0.225, 0.630)]. Compared to adolescents who had known medical problem, the odds of COVID-19-related anxiety was reduced by 59.2% among those who had no known medical problem [AOR (95% CI)], [0.408 (0.278, 0.597)] (Table 4).

**Table 4:** Bivariate and multivariable binary logistic regression results showing factors associated with COVID-19-related dysfunctional anxiety among adolescents in Mettu town, Southwest Ethiopia, September 2020 (*n* = 819)

Variables and category		COVID-19-related anxiety		COR (95% C.I.)	AOR (95% C.I.)
		**Yes**	**No**		
Sex	Male	93 (23.3%	306 (76.7%)	1	1
	Female	78 (18.6%)	342 (81.4%)	0.750 (0.535, 1.052)	0.724 (0.502, 1.043)^*^
Parents	Both or one parent alive	160 (20.1%)	637 (79.9%)	1	1
	Both parents are deceased	11(50%)	11 (50%)	3.981 (1.696, 9.348)	2.981 (1.138, 7.814)^*^
Living status	Lives with parents or others	157 (19.9%)	630 (80.1%)	1	1
	Lives alone	14 (43.8%)	18 (56.3%)	3.121 (1.519, 6.412)	2.363 (1.050, 5.321)^*^
Fathers’ occupation	Employed	40 (16.9%)	197 (83.1%)	01	1
	Unemployed	131 (22.5%)	451 (77.5%)	1.431 (0.967, 2.116)	1.262 (0.805, 1.978)
Mothers’ occupation	Employed	30 ( (15.6%)	162 (84.4%)	1	1
	Unemployed	141 (22.5%)	486 (77.5%)	1.567 (1.017, 2.414)	1.943 (1.194, 3.163)^*^
Fathers’ educational status	No formal education	43 (28.1%)	110 (71.9%)	1	1
	Has formal education	128 (19.2%)	538 (80.8%)	0.609 (0.407, 0.910)	0.709 (0.445, 1.130)
Presence of close friend	No	35 (38%)	57 (62%)	1	1
	Yes	136 (18.7%)	591 (81.3%)	0.375 (0.237, 0.594)	0.377 (0.225, 0.630)^**^
Any known medical problem	Yes	75 (14.6%)	438 (85.4%)	1	1
	No	96 (31.4)	210 (68.6%)	0.375 (0.266, 0.528)	0.408 (0.278, 0.597)^**^
Parents use of substance in the last 3 months	No	151 (19.7%)	614 (80.3%)	1	1
	Yes	20 (37%)	34 (63%)	2.392 (1.339, 4.273)	2.345 (1.217, 4.517)
Substance use in the last 3 months	No	105 (17%)	513 (83%)	1	1
	Yes	66 (32.8%)	135 (67.2%)	2.389 (1.664, 3.429)	2.473 (1.655, 3.694)
Social support	Poor	45 (28.7%)	112 (71.3%)	1	1
	Strong	126 (19%)	536 (81%)	0.585 (0.394, 0.870)	0.705 (0.449, 1.106)
Fear of COVID-19	Low	62 (18.8%)	268 (81.2%)	1	1
	High	109 (22.3%)	380 (77.7%)	1.240 (0.875, 1.757)	1.071 (0.732, 1.565)

Dependent variable: COVID-19-related anxiety

^*^p ≤ 0.05

^**^p < 0.01

^1^Reference

## Discussion

In this study, COVID-19-related dysfunctional anxiety among adolescents and several factors related to this anxiety were evaluated. Accordingly, the magnitude of COVID-19-related dysfunctional anxiety was found to be 20.9% (95% CI (18.1, 23.9)) among adolescents in the study area. Due to the lack of studies conducted among adolescents, comparison with similar studies challenged us. However, the magnitude of COVID-19-related anxiety reported in this study is in line with the magnitude reported in United Kingdom (21.3%) among the general population [31], Bangladesh (23.2%) among older adults [32], and South and Southwest Ethiopia (20.2%) [33] among healthcare professionals. But the findings of the research conducted in Philippines (37.8%) [34], China (70.78%) [35], and Northern Ethiopia (63%) [36] appear to be higher than the magnitude reported in our study. This discrepancy could be attributed to various reasons. The studies in Philippines and Northern Ethiopia were conducted among healthcare workers/nurses who might be more prone to COVID-19-related anxiety since they are involved in the delivery of health care services and directly involved in the care of COVID-19 patients. The study in China was conducted early in the pandemic while lockdown was declared and people in China were overwhelmed with the advent of the fatal pandemic. Further, the study in China evaluated COVID-19-related anxiety using Generalized Anxiety Disorder Scale. Contrary to this, a study conducted in Southeast Ethiopia (16.58%) among residents of the community aged 18 years and above [37], and in Turkey (13.63%) among university students [38] reported lower findings.

In this study, it has been shown that being female adolescent reduces COVID-19-related dysfunctional anxiety. Although there is no another study evaluating this association among adolescents, the research in Bangladesh conducted among older adults [32], Turkey conducted among university students [38], and China conducted among patients [35] showed that females had higher COVID-19 related anxiety scores, which is opposite to the conclusion in our study. The difference may be attributed to the difference in the ways that adolescent females and adult females respond to things causing anxiety. Another possible reason for the discrepancy may be due to the presence of comorbid chronic conditions that may increase anxiety levels of older females compared to adolescent females. Our study has also shown that adolescents who had both of their parents deceased and adolescents who lived alone have higher odds of COVID-19-related dysfunctional anxiety. Our study confirms that having unemployed mothers or lack of close friends during adolescence increases the odds of developing COVID-19-related dysfunctional anxiety. This may be due to the reason that unemployed mothers may not be exposed to crucial health information at workplaces to provide support so that their children can cope with stressors. Furthermore, the study’s finding affirms that absence of known medical problem decreases the odds of developing COVID-19-related dysfunctional anxiety than its presence, and this conclusion is supported by the study conducted in Northern Ethiopia [36], in which presence of chronic diseases predicted higher odds of COVID-19-related anxiety. Patients with comorbid medical conditions are at higher risk of developing severe COVID-19 [39]. Therefore, this could be the possible reason why adolescents with known medical problem experienced higher odds of developing COVID-19-related dysfunctional anxiety in the current study.

This study is the first study in Ethiopia in evaluating COVID-19-related dysfunctional anxiety among adolescents, who are at increased risk of developing mental health problems. Additionally, the study covered the whole age range of adolescence (early, middle, and late-aged adolescents) which increases its representativeness for all adolescent age groups in the study setting. However, the scientific communities should consider some limitations while using the findings of this study. First, there may be introduction of social desirability bias because some of the tools required the study participants to rate themselves on Likert scales. Second, the study could not determine the cause-and-effect relationship because of the nature of the study design used.

## Conclusion

The magnitude of COVID-19-related dysfunctional anxiety was found to be high among adolescents in the study area. The findings have shown that the likelihood of developing COVID-19-related dysfunctional anxiety was linked to several factors. Accordingly, being male, having both parents deceased, living alone, having unemployed mothers, absence of close friends, having known medical problem, substance use, and history of parents’ substance use were associated with COVID-19-related dysfunctional anxiety in adolescents. Therefore, health institutions, nongovernmental organizations, clinicians, and other concerned bodies are encouraged to provide continued psychological support for adolescents during global pandemic like COVID-19. The finding encourages family support services to put out their best efforts in order to ensure that adolescents are healthy. Future researchers are encouraged to investigate the cause-and-effect relationship between different covariates and COVID-19-related dysfunctional anxiety.

## Data Availability

The datasets used and analyzed during the study are available from the corresponding author on reasonable request.

## Abbreviations

AOD: Adjusted odds ratio
CAS: COVID-19 anxiety scale
CI: Confidence interval
COR: Crude odds ratio
COVID-19: Coronavirus disease 2019
ETB: Ethiopian birr
K10: Kessler psychological distress scale
SARS-CoV-2: Severe acute respiratory syndrome coronavirus 2
SD: Standard deviation
SE: Standard error
SPSS: Statistical Product and Service Solution
WHO: World Health Organization

## References

[1] Qiu J (2020). A nationwide survey of psychological distress among Chinese people in the COVID-19 epidemic: implications and policy recommendations. Gen Psychiatr.

[2] COVID-19 pandemic in Africa. https://en.wikipedia.org/wiki/COVID-19_pandemic_in_Africa. Accessed 15 Feb 2023.

[3] WHO. WHO Africa First case of COVID-19 confirmed in Ethiopia. https://www.afro.who.int/news/first-case-covid-19-confirmed-ethiopia. Accessed 5 Jan 2023.

[4] WHO. WHO Coronavirus Disease (COVID-19) Dashboard 2020. 2020. Available from: https://covid19.who.int/?gclid=Cj0KCQiAhs79BRD0ARIsAC6XpaVKVf1AmzsG1TR-1m6f5PGCBlKztaxrroLTtyE3Zr9KJ-uIe8iOkwaAhRTEALw_wcB.

[5] Wang Y (2021). Study on the public psychological states and its related factors during the outbreak of coronavirus disease 2019 (COVID-19) in some regions of China. Psychol Health Med.

[6] Huang Y, Zhao N (2020). Generalized anxiety disorder, depressive symptoms and sleep quality during COVID-19 outbreak in China: a web-based cross-sectional survey. Psychiatry Res.

[7] Wu Y (2020). Nervous system involvement after infection with COVID-19 and other coronaviruses. Brain Behav Immun.

[8] Desforges M (2019). Human coronaviruses and other respiratory viruses: underestimated opportunistic pathogens of the central nervous system?. Viruses.

[9] Dantzer R (2018). Neuroimmune interactions: from the brain to the immune system and vice versa. Physiol Rev.

[10] Netland J (2008). Severe acute respiratory syndrome coronavirus infection causes neuronal death in the absence of encephalitis in mice transgenic for human ACE2. J Virol.

[11] Brooks SK (2020). The psychological impact of quarantine and how to reduce it: rapid review of the evidence. Lancet.

[12] de Medeiros Carvalho PM (2020). The psychiatric impact of the novel coronavirus outbreak. Psychiatry Res.

[13] WHO. The health of youth. Geneva. 1989. (document A42/Technical Discussions/2).

[14] Casey B (2010). The storm and stress of adolescence: insights from human imaging and mouse genetics. Dev Psychobiol.

[15] Bailen NH, Green LM, Thompson RJ (2019). Understanding emotion in adolescents: a review of emotional frequency, intensity, instability, and clarity. Emot Rev.

[16] Somerville LH, Jones RM, Casey B (2010). A time of change: behavioral and neural correlates of adolescent sensitivity to appetitive and aversive environmental cues. Brain Cogn.

[17] Somerville LH (2013). The teenage brain: sensitivity to social evaluation. Curr Dir Psychol Sci.

[18] Meuwese R, Cillessen AH, Güroğlu B (2017). Friends in high places: a dyadic perspective on peer status as predictor of friendship quality and the mediating role of empathy and prosocial behavior. Soc Dev.

[19] La Greca AM, Harrison HM (2005). Adolescent peer relations, friendships, and romantic relationships: do they predict social anxiety and depression?. J Clin Child Adolesc Psychol.

[20] Golberstein E, Wen H, Miller BF (2020). Coronavirus disease 2019 (COVID-19) and mental health for children and adolescents. JAMA Pediatr.

[21] Ali MM (2019). Utilization of mental health services in educational setting by adolescents in the United States. J Sch Health.

[22] Ellis WE, Zarbatany L (2017). Understanding processes of peer clique influence in late childhood and early adolescence. Child Dev Perspect.

[23] Danese A (2009). Adverse childhood experiences and adult risk factors for age-related disease: depression, inflammation, and clustering of metabolic risk markers. Arch Pediatr Adolesc Med.

[24] Jones PB (2013). Adult mental health disorders and their age at onset. Br J Psychiatry.

[25] Stephanie G. Kish Grid: what it is and how to use it‖ from StatisticsHowTo.com: elementary statistics for the rest of us!. 2017. Available from: https://www.statisticshowto.com/kish-grid/.

[26] Abiola T, Udofia O, Zakari M (2013). Psychometric properties of the 3-item Oslo social support scale among clinical students of Bayero University Kano, Nigeria. Malays J Psychiatry.

[27] Ahorsu DK, et al. The fear of COVID-19 scale: development and initial validation. Int J Mental Health Addict. 2020:20(3):1–9. 10.1007/s11469-020-00270-8 PMC7100496 32226353

[28] Abdisa DK (2022). Access to maternal health services during COVID-19 pandemic, re-examining the three delays among pregnant women in Ilubabor zone, southwest Ethiopia: a cross-sectional study. PLoS ONE.

[29] Lee SA (2020). Coronavirus anxiety scale: a brief mental health screener for COVID-19 related anxiety. Death Stud.

[30] Hosmer DW Jr, Lemeshow S, Sturdivant RX. Applied logistic regression. Vol. 398. Geneva: Wiley; 2013.

[31] Shevlin M (2020). Anxiety, depression, traumatic stress and COVID-19-related anxiety in the UK general population during the COVID-19 pandemic. BJPsych Open.

[32] Mistry SK (2022). COVID-19 related anxiety and its associated factors: a cross-sectional study on older adults in Bangladesh. BMC Psychiatry.

[33] Alenko A (2021). COVID-19-related anxiety and its association with dietary diversity score among health care professionals in Ethiopia: a web-based survey. J Multidiscip Healthc.

[34] Labrague LJ, De los Santos JAA (2020). COVID-19 anxiety among front-line nurses: predictive role of organisational support, personal resilience and social support. J Nurs Manag.

[35] Tang F (2021). COVID-19 related depression and anxiety among quarantined respondents. Psychol Health.

[36] Kibret S (2020). Prevalence of anxiety towards COVID-19 and its associated factors among healthcare workers in a Hospital of Ethiopia. PLoS ONE.

[37] TeferuEngida Z (2021). COVID-19-related anxiety and the coping strategies in the Southeast Ethiopia. Psychol Res Behav Manag.

[38] Durbas A (2021). Anxiety and stress levels associated with COVID-19 pandemic of university students in Turkey: a year after the pandemic. Front Psych.

[39] Liu H (2020). Comorbid chronic diseases are strongly correlated with disease severity among COVID-19 patients: a systematic review and meta-analysis. Aging Dis.

